# College students’ psychological resilience and daily recovery: a narrative review

**DOI:** 10.3389/fpsyg.2026.1944087

**Published:** 2026-09-10

**Authors:** Chao Li

**Affiliations:** Henan Medical University, Xinxiang, Henan, China

**Keywords:** conservation of resources theory, daily recovery, interpersonal safety, perceived academic control, psychological resilience, sleep rhythm

## Abstract

Research on college students’ psychological resilience has long focused on stable protective and risk factors at the individual level, while paying insufficient attention to the recovery processes in response to daily micro-stressful contexts. Framed by the Conservation of Resources theory, this narrative review reconceptualizes resilience as the long-term outcome of sustained successful adaptation to everyday demands, a capacity that a daily recovery process helps to build and maintain. It specifies this process across three resources: sleep rhythm, daily interpersonal safety, and perceived academic control. This synthesis clarifies their recovery mechanisms, sources of vulnerability, and reinforcing pathways. On this basis, a three-dimensional daily recovery conceptual framework is proposed, which delineates two pathways of gain and loss dynamics and two temporal patterns of immediate transmission and cumulative trajectory. The framework shifts the research focus from static predictors of resilience to the daily conditions under which recovery occurs, and accordingly redirects intervention priorities toward providing systemic contextual supports that enable, rather than replace, the cultivation of individual capabilities.

## 1 Introduction

Much of the research on college students’ psychological resilience has taken major adversity as a primary context (Bonanno, 2004) and measured resilience as a stable individual difference through cross-sectional scales (Connor and Davidson, 2003; Luthar et al., 2000; Masten, 2001). The trait-focused perspective has three limitations for explaining daily psychological adaptation. Methodologically, such scales capture only static scores at a single time point (Wu et al., 2020), failing to reflect dynamic changes in daily stress contexts (Child and Medvedev, 2024). In focus, research has centered on major adversities such as childhood trauma (Kelifa et al., 2020; Chang et al., 2021), yet most students face not extreme adversity but chronic resource depletion from daily academic stress and interpersonal friction (Barbayannis et al., 2022; Gong et al., 2023). In intervention, programs based on the adversity-resistance paradigm have yielded small effect sizes (Houston et al., 2017) and remain disconnected from students’ daily rhythms. These limitations call for a recovery-centered, dynamic perspective.

To address these limitations, this article draws on conservation of resources (COR) theory to reconceptualize resilience as the long-term outcome of sustained successful adaptation to everyday demands, a capacity that a daily recovery process helps to build and maintain. This process involves three dimensions: physiological, social, and cognitive, and each is expressed through one representative resource: sleep rhythm, daily interpersonal safety, and perceived academic control. Throughout this review, the three resources are treated not as resilience itself, but as resources that support daily recovery. COR theory holds that resources are self-reinforcing and cumulative: those with scarce resources may fall into loss dynamics, whereas the resource-rich may initiate gain dynamics (Hobfoll, 1989, 2002). COR theory further holds that resources travel together as interconnected bundles, so that a gain or loss in one resource tends to affect the others (Hobfoll, 2011). Within this framework, the path from depletion to resilience comprises four steps. First, daily micro-stressors deplete resources in the physiological, social, and cognitive dimensions, that is, resource depletion. Second, recovery replenishes these resources, with their replenished levels serving as resource indicators of the current state. Third, once replenished, individuals can maintain everyday functioning, that is, short-term adaptation. Fourth, when replenishment occurs repeatedly and stably, individuals come to sustain successful adaptation over the long term; this sustained successful adaptation constitutes resilience, with daily recovery functioning as its underlying mechanism rather than its definition (Masten, 2014).

This framework differs from several related approaches. Academic buoyancy describes students’ ability to deal with everyday academic setbacks, but its scope remains confined to the academic domain (Martin and Marsh, 2008). Recovery theory addresses recovery from work and rests on a clear work–nonwork separation, whereas stressors on campus are dispersed throughout the day without such boundaries (Sonnentag and Fritz, 2007). Dynamic resilience models treat resilience as a dynamic system’s adaptive capacity, but remain largely conceptual and do not specify observable daily indicators (Masten, 2014). The present framework integrates these separate strands into a three-dimensional recovery framework oriented toward everyday campus stressors.

## 2 Methods

This review adopts a narrative review approach suited to critical reading, thematic integration, and extracting conceptual insights from methodologically heterogeneous literature rather than exhaustive evidence synthesis (Ferrari, 2015). PubMed, Web of Science, and Scopus were searched from 1985 to June 2026. Search terms included: (“psychological resilience” OR “resilience” OR “daily recovery” OR “recovery experience”) AND (“college student*” OR “university student*” OR “undergraduate*”) AND (“sleep rhythm” OR “circadian rhythm” OR “interpersonal relationship” OR “social support” OR “dormitory” OR “academic control” OR “perceived control”). The asterisk (*) was used as a wildcard to expand search terms where applicable.

Inclusion criteria were: (a) participants were college students or young adults aged 18 to 25; as predefined exceptions, studies whose samples were predominantly college students but with slightly wider age ranges, and experimental studies that used adult samples to provide causal mechanistic evidence, were retained, (b) the study addressed at least one of the three resources—sleep rhythm, interpersonal safety, or perceived academic control, and (c) the article was a peer-reviewed English-language journal publication. The initial literature search yielded 656 records. After removing duplicates, excluding clinical inpatient samples, and reviewing titles, abstracts, and full texts, 50 studies met the inclusion criteria and were included in this review. Theoretical and methodological sources are cited as background literature to frame the analysis but are not counted among these included studies. Representative studies, selected for recency, rigor, and illustrative capacity, are listed in Table 1 for each resource.

**Table 1:** Selected studies by recovery resource.

Resource	Study (Country)	Sample (*N*; age)	Design	Key finding (with estimate)	Limitation
3.1	Meng et al., 2025 (China)	343; healthcare students	Cross-lagged panel	Sleep quality predicted lower internalizing symptoms via reduced perceived stress (*β* = 0.017, *p* = 0.038)	Self-report; single-site sample
	Ghoul et al., 2025 (United States)	108; undergraduates; 18–24 y	RCT	Brief sleep hygiene intervention improved sleep quality (ηp^2^ = 0.21) and reduced stress (ηp^2^ = 0.07)	Single-site; short follow-up
	Eker and Yıldırım, 2022 (Turkey)	1,040; university students; 18–22 y	Cross-sectional	Chronotype was associated with resilience; evening types lower (*R*^2^ = 0.219)	Cross-sectional; causal direction unclear
	Li M. et al., 2022 (China)	364; college students; M = 20.1 (1.3)	Cluster RCT	Dormitory sleep rules and environmental optimization improved sleep quality (*β* = −0.67, *p* = 0.012)	Single-site; short follow-up
3.2	Pan et al., 2025 (China)	1,408; college students; M = 18.94 (1.01)	Latent profile analysis	Avoidant/competitive conflict coping (15%) associated with higher depression/anxiety	Cross-sectional; causal direction unclear
	Chen et al., 2026 (China)	3,765; college students	Longitudinal	Interpersonal sensitivity mediated the interpersonal stress–loneliness link (within-person; females)	Self-report; single country
	Li et al., 2025 (14 countries)	51 studies	Systematic review	Social support was associated with better wellbeing via direct and indirect effects	Heterogeneous evidence
	Xu et al., 2025 (China)	601; medical students	Cross-sectional	Dormitory interpersonal atmosphere associated with academic performance [OR = 1.60, 95% CI (1.10, 2.31)]	Cross-sectional; causal direction unclear
3.3	Luo et al., 2024 (China)	679; college students; 18–25 y	Cross-sectional	Attributing outcomes to effort/ability associated with lower depression risk (OR = 0.90, 95% CI [0.83, 0.97])	Cross-sectional; causal direction unclear
	Wen and Jin, 2025 (China)	730; college students; M = 20.37 (1.94)	Cross-sectional	Upward social comparison associated with lower control via self-esteem (*β* = −0.21) and stress (*β* = 0.21)	Cross-sectional; causal direction unclear
	French et al., 2024 (Multi-country)	—	Systematic review	High-stakes exams associated with lower intrinsic motivation and elevated stress markers	Heterogeneous evidence
	Dryden et al., 2021 † (Canada)	765; first-generation college students; 17–20 y	RCT	Attributional retraining improved grades (B vs. C+) and reduced course drop by 55%	Single-site; short follow-up
	Liu, 2025 (China)	381; college students; M = 20.7 (2.3)	Cross-sectional (mixed)	Growth mindset associated with greater academic buoyancy via resilience (*β* = 0.21, *p* = 0.004)	Cross-sectional; causal direction unclear
	van der Mee et al., 2026 † (Netherlands)	152; college students; 18–55 y	EMA	Negative affect rose during exams then recovered; positive affect continued to decline	Short assessment window; single-site
Cross-domain	Gordon-Hecker et al., 2025 ‡ (USA & UK)	155 + 347; adults; M = 36.99/35.49	Experimental	Sleep loss was associated with reduced empathy (*r* = 0.20, *p* = 0.014)	Adult convenience sample
	Gu et al., 2026 (China)	417; college students; M = 18.28 (0.90)	Longitudinal (21-day)	Sleep health and rumination showed dynamic reciprocal relations	Self-report; single country
	Soon and Chua, 2025 (Singapore)	638; college students; M = 20.3 (1.3)	Longitudinal	On-campus students had shorter weekday sleep than off-campus students (−19.03 min, *t* = 6.36, *p* < 0.0001)	Single-site sample
	Dong et al., 2025 † (China)	1,065; college students; M = 19.56	Cross-sectional	Loneliness associated with poorer sleep via stress and expressive suppression (direct *β* = 0.097; chain mediation)	Cross-sectional; causal direction unclear
	Li, 2024 (China)	1,085; college students	Cross-sectional	Peer support associated with better sleep via physical exercise atmosphere (*β* = −0.214)	Cross-sectional; causal direction unclear
	Xiang et al., 2026 (China)	741; college students; M = 19.46 (1.49)	Cross-sectional	Academic stress associated with bedtime procrastination via rumination (*β* = 0.24)	Cross-sectional; causal direction unclear

M, mean age; SD, standard deviation; y, years. Studies are listed as representative evidence for each recovery domain rather than an exhaustive list of the 50 included articles. † denotes studies with slightly wider age ranges whose samples were predominantly college students; ‡ denotes experimental studies using adult samples to provide causal mechanistic evidence.

## 3 Three recovery resources: evidence synthesis through the Lens of COR theory

The recovery experience model of Sonnentag and Fritz (2007, 2015) identifies four recovery experiences: psychological detachment, relaxation, mastery, and control, but is grounded in employees’ work-nonwork binary context. Campus differs in this respect, so sleep rhythm, interpersonal safety, and perceived academic control were selected as the three resources, which correspond to the physiological, social, and cognitive dimensions supporting daily recovery. Because this grouping is a synthesis rather than an exhaustive classification, it is regarded as a proposed framework.

### 3.1 Sleep rhythm: the physiological condition for net resource gain

Sleep constitutes the physiological resource of college students’ daily recovery. Within the COR framework, adequate sleep serves as the core cyclical mechanism of net resource gain, with each episode of sufficient sleep systematically replenishing the day’s depleted cognitive and emotional resources. A cross-lagged panel study showed that sleep quality predicted lower internalizing symptoms via reduced perceived stress (Meng et al., 2025); a randomized controlled trial showed that a brief sleep hygiene intervention improved sleep quality and thereby reduced stress (Ghoul et al., 2025). At the neuroendocrine level, this recovery process is associated with the stability of the cortisol circadian rhythm, whereas circadian disruption can interfere with emotion-regulation circuits and increase depression and anxiety risk (Dollish et al., 2024). Maintaining regular sleep rhythm is thus an important recovery mechanism for college students’ daily resilience.

College students face unique sleep vulnerabilities: social jetlag (the weekday–weekend sleep schedule discrepancy; Lopes et al., 2022), sleep debt accumulation during exam periods (Suardiaz-Muro et al., 2023), and nighttime environmental disturbances in dormitories (Wang et al., 2023) systematically compress the physiological recovery window. Among 1,040 Turkish university students, chronotype was significantly associated with resilience, with evening-type students scoring lower, suggesting that chronotype may moderate daily recovery (Eker and Yıldırım, 2022). Multiple studies have shown more pronounced sleep problems in female college students (Becker et al., 2018; Putilov et al., 2021), suggesting sex differences relevant to targeted intervention design.

However, campus intervention research incorporating sleep into daily recovery remains emergent (Tadros et al., 2025). Dormitory sleep rules and light environment optimization improve sleep quality (Li M. et al., 2022), while digital cognitive behavioral therapy (Li X. et al., 2022) and mindfulness-based cognitive therapy (Hao et al., 2026) have shown positive effects. A randomized controlled trial showed that a four-week bedtime sound intervention improved sleep quality and reduced perceived stress (Hou et al., 2026). These findings indicate that low-cost sleep rhythm regulation strategies hold potential for campus-wide implementation.

### 3.2 Daily interpersonal safety: the protective and buffering functions of social support

Within the COR framework, social relationships are both a personal resource and a passageway for resources flow (Hobfoll, 2002). College students’ daily interpersonal safety rests on two settings: dormitory co-residence with long-term and frequent roommate contact, and peer interaction beyond the dormitory, such as club participation and friendships. Empirical research indicates that social support is positively associated with college students’ resilience, with coping styles mediating this association (Cao et al., 2024). Social support plays a dual role: a high-quality dormitory relation provides daily protection for resource maintenance (a main effect; Xu et al., 2025), whereas peer support offers timely buffering when acute depleting situations arise (a buffering effect; Cohen and Wills, 1985). Daily interpersonal safety thus serves both protective and remedial functions in the daily recovery cycle.

However, the interpersonal environment of college students may undermine these functions. As a co-living space, dormitories cannot entirely avoid interpersonal conflict. A latent profile analysis found that approximately 15% tended toward avoidant or competitive conflict coping, associated with higher depression, anxiety, and stress (Pan et al., 2025). Furthermore, interpersonal stress does not affect all students uniformly. A two-year longitudinal study of 3,765 Chinese college students found that interpersonal sensitivity mediated the link between interpersonal stress and loneliness (Chen et al., 2026). Moreover, the protective benefits of social support are not automatic. A systematic review found that social support contributes to college students’ wellbeing through direct and indirect effects via enhanced resilience and self-esteem (Li et al., 2025). Taken together, college students’ interpersonal environment may face threefold constraints: structural conflict, sensitivity differences, and insufficient initiative.

Building on these findings, strengthening daily interpersonal safety can proceed along three mutually complementary directions. Preventing dormitory conflict should not rely solely on individuals’ interpersonal skills; dormitory-level rule design helps reduce interpersonal depletion at the source (Li M. et al., 2022). Individual differences in interpersonal sensitivity imply that interventions require precision: prioritizing highly sensitive students with targeted support may be more effective than universal interpersonal education (Chen et al., 2026). The protective function of social support depends on perceived support availability; cultivating positive cognition about social relationships may activate this function more effectively than teaching social skills (Imwinkelried et al., 2025; Li et al., 2025). Daily interpersonal safety should thus be built into the campus recovery system.

### 3.3 Perceived academic control: the regulatory pathway of cognitive depletion

Within the COR framework, sense of control is a special resource. It not only buffers stress-induced depletion but also determines whether other resources can be effectively mobilized (Hobfoll, 2002). Daily academic stress is among the most frequent sources of resource depletion for college students. A 2024 national survey found that 76.4% of college students reported moderate to high levels of stress (American College Health Association, 2024), and academic stress was the most frequent antecedent of psychological distress (Roy et al., 2025). Perceived academic control is the core cognitive variable regulating this depletion (Perry et al., 2001). Attribution theory reveals that attributing setbacks to controllable factors buffers subsequent depletion of cognitive resources, whereas attributing them to uncontrollable factors accelerates resource loss through learned helplessness (Weiner, 1985). Cross-lagged longitudinal research showed that self-efficacy beliefs reduced subsequent perceived stress (Liu et al., 2024). Attributional retraining research demonstrates the plasticity of this mechanism: correcting maladaptive attributions reduces the risk of course failure and restores sense of control (Haynes-Stewart et al., 2011).

Yet college students’ perceived academic control faces systematic erosion in daily life. High-stakes examinations are associated with distress, anxiety and elevated physiological stress markers, continuously depleting cognitive and emotional resources (French et al., 2024). Uncontrollable attribution intensifies this erosion: attributing outcomes to effort or ability was associated with lower depression risk, whereas the absence of such controllable attributions leaves students more vulnerable (Luo et al., 2024). Competitive evaluative environments pose a further threat: upward social comparison is associated with lower sense of control via lowered self-esteem and increased perceived stress (Wen and Jin, 2025). These erosive effects are not evenly distributed: senior and medical students face higher risk (Luo et al., 2024). A 28-day EMA study showed that negative affect increased during exam periods and then recovered, whereas positive affect continued to decline during the recovery period (van der Mee et al., 2026), suggesting temporally delayed cognitive depletion. In contrast, growth mindset is associated with greater academic buoyancy via strengthened psychological resilience (Liu, 2025).

Building on these findings, strengthening perceived academic control requires coordinated efforts across three directions: the assessment system, attributional training, and group differences. At the assessment-system level, shifting from high-stakes summative examinations to distributed, low-stakes formative assessment is associated with reduced evaluation anxiety and greater psychological sense of control (McGuire, 2026). In attribution training, a randomized trial showed that students guided to reframe setbacks from uncontrollable to controllable attributions improved final grades by a letter grade and reduced the likelihood of dropping the course by 55% among low-control first-generation students, relative to those who received no retraining (Dryden et al., 2021). At the level of group differences, attributional retraining is particularly effective for low-control students (Dryden et al., 2021), suggesting that intervention resources should be directed preferentially toward high-risk groups. Perceived academic control should therefore be treated as a systemic condition.

## 4 The interplay among the three recovery resources

**Figure 1:**
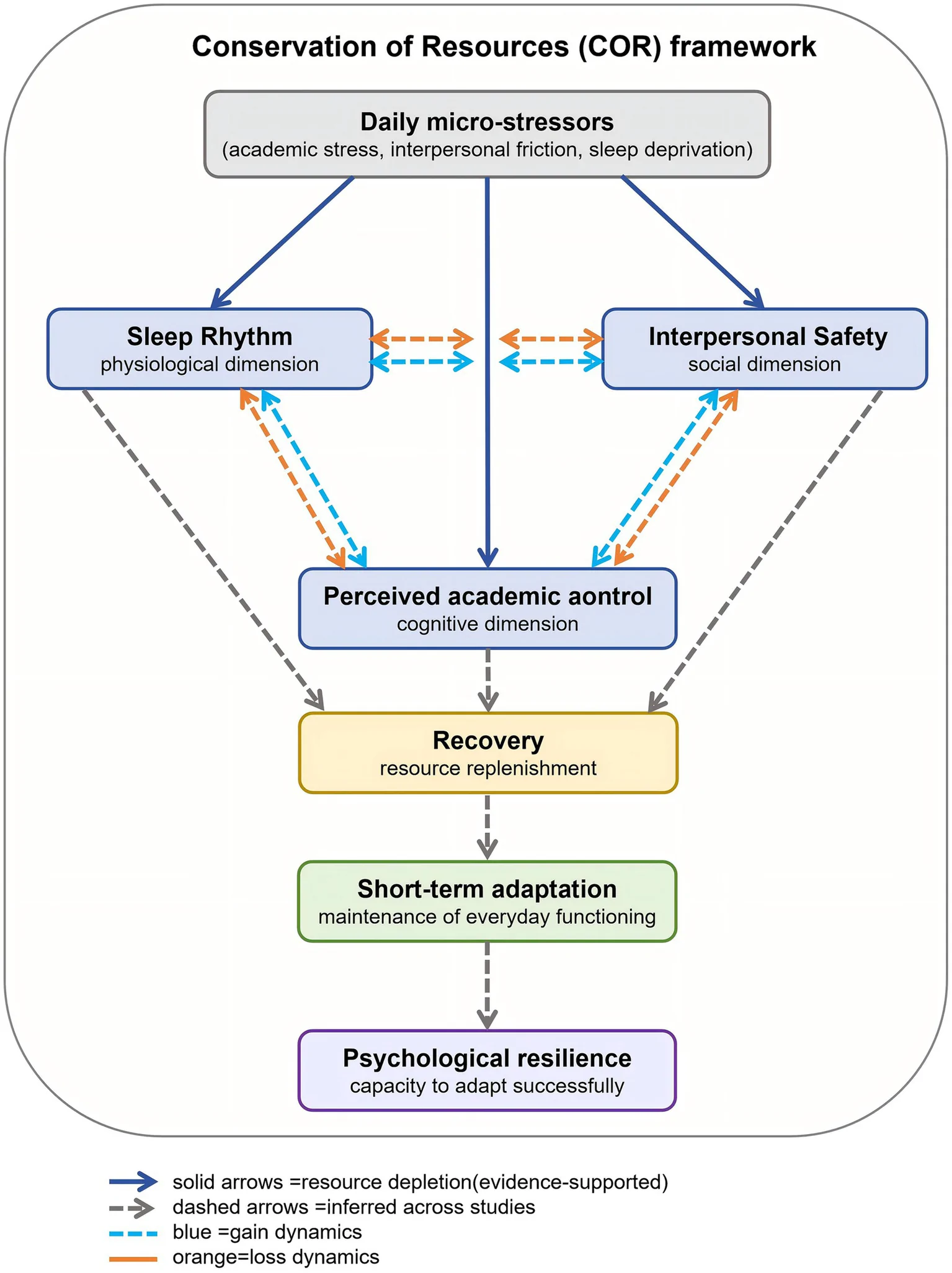
A three-dimensional daily recovery conceptual framework of college students’ psychological resilience within the Conservation of Resources (COR) framework. Daily micro-stressors (academic stress, interpersonal friction, and sleep deprivation) deplete the three resources (sleep rhythm, interpersonal safety, and perceived academic control) across physiological, social, and cognitive dimensions (solid arrows, supported by evidence and established theory). The three resources are interconnected through gain dynamics (blue) and loss dynamics (orange); these cross-domain pathways and the downstream pathways (dashed arrows) are inferred across separate studies. The two temporal patterns of this interplay, immediate transmission and cumulative trajectory, are detailed in the main text. The three dimensions support recovery, the replenishment of resources; sustained recovery supports short-term adaptation, the maintenance of everyday functioning, which in turn sustains resilience.

The three resources constitute an interconnected system through gain dynamics and loss dynamics (Figure 1). Along the gain pathway, well-rested individuals display greater helping willingness and empathic capacity, fostering interpersonal safety (Ben-Simon et al., 2022; Gordon-Hecker et al., 2025); peer support is associated with better sleep quality (Li, 2024), and sleep quality and rumination mutually reinforce to form a gain cycle (Gu et al., 2026). Perceived academic control serves resource-protective and resource-mobilizing functions: teacher feedback and peer social support are key external resources (Wang et al., 2025; Yu et al., 2026). Along the loss pathway, a low level in any resource may trigger a chain of resource erosion. Sleep debt accumulation is predicted to weaken emotion regulation and intensify interpersonal friction (Hobfoll, 2002); uncontrollable academic stress may, via rumination, lead to bedtime procrastination and disrupt sleep rhythm (Xiang et al., 2026; Cong et al., 2024); and interpersonal alienation is associated with poorer sleep via perceived stress and expressive suppression, suggesting that blocked social channels may indirectly weaken recovery (Dong et al., 2025). Together, these pathways show that the three recovery resource bases are interdependent.

The interplay among the three resources exhibits two temporal patterns: immediate transmission and cumulative trajectory. Immediate transmission is operationalized as within-person, same-day or next-day lagged effects between resources (e.g., a 24-h time lag in EMA designs). Consistent with COR theory’s resource self-reinforcement principle and with sleep-interpersonal evidence (Ben-Simon et al., 2022; Gordon-Hecker et al., 2025), the author proposes that sleep quality of a given night predicts next-day interpersonal safety and academic control. If no same-day or next-day lagged association emerges across an academic term, even during high-demand weeks, this proposition would be falsified. This transmission may operate directly or indirectly across the three resources. This transmission is proportional to the magnitude of the sleep change, such that single-night mild changes yield effects too small to be detected in typical EMA samples. Rumination has been implicated as a mediating mechanism in the stress-sleep link, consistent with the proposed immediate transmission (Zhang et al., 2024). Cumulative trajectory is operationalized as the rate of change in focal variables over an extended period, reflecting net resource accumulation or loss over time. Drawing on COR’s loss/gain spiral concept, the author proposes that when immediate transmission recurs, its effects accumulate into loss and gain spirals. Depletion in one resource weakens the others, so that losses spread; conversely, recovery in one resource strengthens the others, so that gains amplify. Whether this accumulation occurs, and in which direction, depends on the continuity of recovery opportunities: if students can continuously access recovery opportunities, short-term losses are replenished in time; only when such opportunities are chronically absent do losses accumulate and the loss spiral accelerate. Residential students slept less as evening socializing encroached on rest time, tentatively suggesting cross-resource dynamics (Soon and Chua, 2025).

## 5 Practical implications and future directions

The maintenance of daily resilience is the result of sustained positive interplay among three resources: sleep, interpersonal, and academic. Campus mental health intervention should optimize the recovery system while continuing to enhance individual capacity, treating systemic supports as the resource passageways through which individual capacity is exercised and sustained. Existing evidence points to three actionable directions: dormitory sleep rules and environmental optimization improved sleep (Li M. et al., 2022); peer support programs can enhance new students’ sense of school belonging (Costello et al., 2022); and distributed assessment and attributional retraining provide feasible pathways for enhancing perceived academic control (Dryden et al., 2021; McGuire, 2026). These directions are best implemented as a hybrid: systemic changes widen ecological passageways, while attributional retraining equips students to use them, so structural reform and individual autonomy reinforce one another. These directions draw mainly on evidence from residential college students, and their broader applicability awaits examination.

Several limitations of this review should be noted. As a narrative review aimed at theoretical construction, the inclusion and synthesis of the literature inevitably involved selective judgment. The pathways of the three-dimensional dynamics are currently supported mainly by indirect evidence from pairs of resources, and direct studies examining all three resources simultaneously remain lacking. The evidence base draws heavily on Chinese, residential, and medical-student samples, and its coverage of higher education systems and cultural contexts is uneven; the framework’s applicability to student populations that differ in living arrangements, study modes, and life circumstances therefore awaits examination.

Future studies could test this framework through an EMA design spanning an academic term. Daily sleep quality could be recorded via wearable devices or sleep diaries, dormitory social interactions via self-reports, and perceived academic workload via time- or event-contingent prompts. These repeated measures would allow cross-resource stress transmission to be examined through multilevel and cross-lagged models. Complementing this observational arm, a randomized controlled trial could test the real-world effectiveness of the proposed intervention directions. Among high-risk groups, a dormitory conflict-management intervention is hypothesized to improve sleep quality and diurnal cortisol rhythm, and increase academic self-efficacy. This hypothesis is generated by the present framework rather than established by the reviewed evidence. The author proposes that cross-resource transmission is stronger among higher-risk students and during high-demand periods. Overall, the core propositions of the framework await examination through longitudinal designs and randomized controlled trials.

## 6 Conclusion

This review has reconceptualized college students’ psychological resilience as the long-term outcome of sustained successful adaptation to everyday demands over time, a capacity that a daily recovery process helps to build and maintain: individual resources are continuously depleted under daily micro-stressors, and sleep rhythm, interpersonal safety, and perceived academic control constitute the three resources that support recovery. Sleep rhythm drives physiological replenishment, interpersonal safety provides social buffering, and perceived academic control regulates cognitive depletion. These three resources do not operate independently; gain dynamics enable mutual reinforcement, whereas loss dynamics may lead to mutual depletion. These dynamics are reflected as immediate transmission in the short term and cumulative trajectory in the long term. With COR theory as the analytical framework, the framework shifts the focus from “static predictors of resilience” to “the conditions that make recovery possible”, and accordingly reorients intervention toward the provision of systemic conditions that support, rather than replace, individual capacity building.
